# Serotonylation: Serotonin Signaling and Epigenetics

**DOI:** 10.3389/fnmol.2019.00288

**Published:** 2019-11-21

**Authors:** Michael Bader

**Affiliations:** ^1^Max-Delbrück Center for Molecular Medicine, Berlin, Germany; ^2^Institute for Biology, University of Lübeck, Lübeck, Germany; ^3^Charité University Medicine, Berlin, Germany; ^4^German Center for Cardiovascular Research (DZHK), Partner Site Berlin, Berlin, Germany

**Keywords:** serotonin, histone, G-protein, protein modification, pulmonary hypertension, epigenetics, monoaminylation, transglutaminase

## Abstract

Serotonylation, the covalent linkage of serotonin to proteins has been discovered more than 60 years ago but only recently the mechanisms and first functions have been elucidated. It has been found that transglutaminases (TG) such as TG2 and the blood coagulation factor XIIIa are the enzymes which catalyze the linkage of serotonin and other monoamines to distinct glutamine (Gln) residues of target proteins. The first target proteins, small G-proteins and extracellular matrix constituents, were found in platelets and are pivotally involved in platelet aggregation and the formation of thrombi. The serotonylation of the same proteins is also involved in insulin secretion and in the proliferation of pulmonary vascular smooth muscle cells and thereby in the pathogenesis of pulmonary arterial hypertension (PAH). Recently histones have been described as targets of serotonylation opening the area of transcriptional control to this posttranslational protein modification. Future studies will certainly reveal further target proteins, signaling pathways, cellular processes, and diseases, in which serotonylation or, more general, monoaminylation is important.

## Introduction in Transglutamination

**FIGURE 1:**
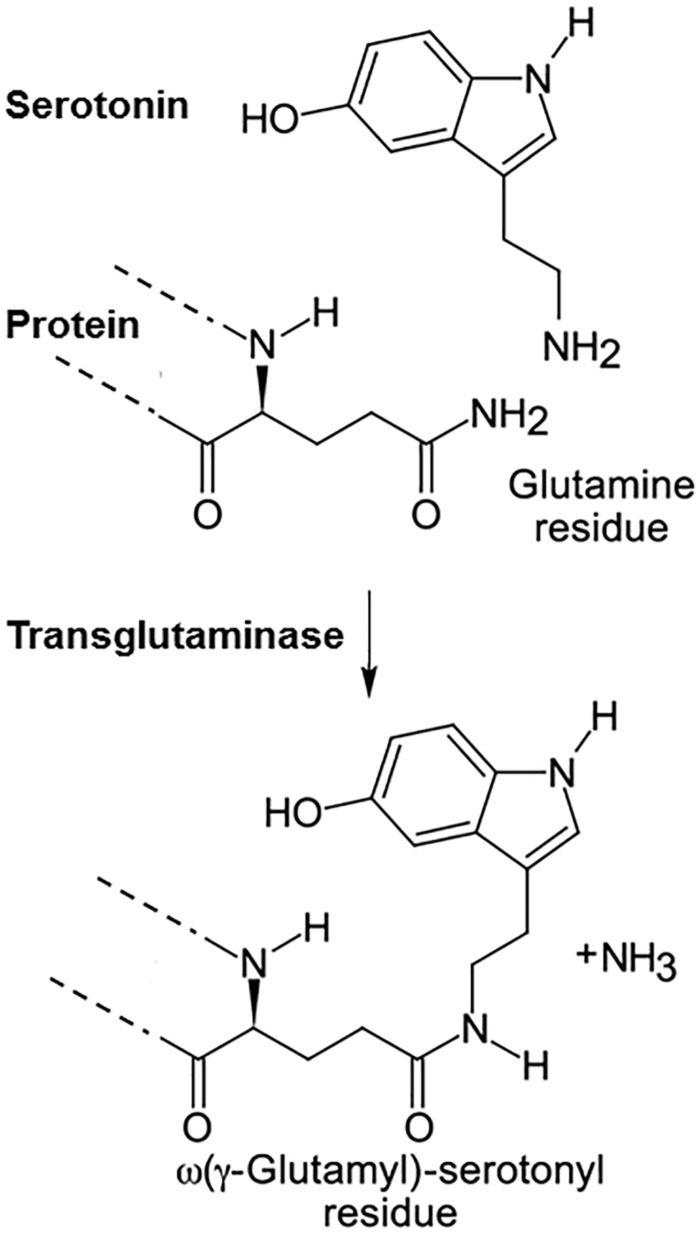
Serotonylation. Serotonin is covalently linked to glutamine residues in proteins by transglutaminases.

In the late 1950s the group of Waelsch et al. discovered that primary amines can be incorporated into proteins by being covalently linked to glutamine (Gln) residues (Sarkar et al., 1957). These included besides polyamines also monoamines such as serotonin, histamine and norepinephrine (Clarke et al., 1959). This group also discovered the enzyme catalyzing this reaction and coined the term transglutaminase for it (Mycek et al., 1959). However, the function of this posttranslational modification of proteins remained unclear for more than 30 years. In the meantime at least 8 different transglutaminases (TG) were described, TG1-7 and blood coagulation factor XIII, and it was shown that these enzymes also crosslink proteins by linking lysine residues of one protein to glutamines of another (Lorand and Graham, 2003; Eckert et al., 2014; Lai et al., 2017). In contrast to TGs, which are mainly intracellular, factor XIII is extracellular and needs to be activated to factor XIIIa by thrombin during coagulation. TG can stiffen extracellular matrix by “gluing” proteins together and therefore such enzymes are used in the food industry to process meat. The first hint on the function of poly- or monoaminylation of proteins came from studies on bacterial toxins, such as Bordetella toxin, which are also transglutaminases. Intracellularly, they transamidate polyamines to small G-proteins and thereby modulate the immune response of the host (Aktories, 2011). In most cases infectious agents hijack cellular processes and redirect them for their purpose. Thus when we looked for the mechanism by which serotonin regulates platelet aggregation we considered that it could do this via the mechanism the bacterial toxins exploited. Indeed, we discovered that serotonin is covalently bound to small G-proteins in the cytoplasm by the endogenous transglutaminase TG2 (Walther et al., 2003). For this process we coined the term serotonylation (Figure 1). Already one year earlier Dale et al. had discovered that serotonin can be covalently linked to fibronectin and other extracellular proteins of platelets by factor XIIIa, which generates a particularly active form of thrombocytes, called coated platelets (Dale et al., 2002; Dale, 2005).

TG2 is the most likely candidate for serotonylation in most organs, since it is widely expressed in contrast to most other members of the TG family (Lorand and Graham, 2003; Eckert et al., 2014; Muma and Mi, 2015) and its reduction using siRNA nearly completely inibited Rac1 serotonylation in neuronal cells (Dai et al., 2008). Moreover TG1 and TG3 could not transamidate small G-proteins (Vowinckel et al., 2012). However, other TGs may anyhow contribute at least in some tissues since for example in aortic smooth muscle cells the specific inhibitor for TG2, Z-DON, reduced transamidation less efficiently than the general TG inhibitor, cystamine (Johnson et al., 2012).

TG2 exhibits a very peculiar way of activation (Katt et al., 2018). It has binding sites for the guanine nucleotides, GTP or GDP, and for calcium which are mutually exclusive. At normal intracellular concentrations of GTP or GDP TG2 remains in a very compact and inactive configuration and only when intracellular calcium concentrations rise e.g., elicited by the activation of cell surface receptors the enzyme adopts the open state and can transamidate substrates. It is not well understood how substrate specificity and the selection of the modified Gln residues is achieved. Accessibility of the Gln residue for the enzyme active site seems to be a major determinant but a specific serotonylation motif has not yet been defined (Lorand and Graham, 2003; Lai et al., 2017).

## Methods to Detect Serotonylation

### Direct Measurement of Serotonin

Dale et al. (2002) purified the serotonylated protein, in their case fibrinogen, hydrolyzed the bond with serotonin using mercaptoethane sulfonic acid and detected serotonin by HPLC. This method however, only works when the protein is present in high amounts and can be easily purified.

### Radioactive Serotonin

The most obvious method to detect the covalent modification of proteins by serotonin is the use of ^3^H- or ^14^C-labeled serotonin (Walther et al., 2003; Paulmann et al., 2009; Hummerich et al., 2012; Al-Zoairy et al., 2017; Farrelly et al., 2019). Measuring radioactivity in protein precipitates or autoradiography of protein gels reveals the incorporation of the monoamine in certain proteins. However, this method is not very sensitive due to the weak radioactivity of ^3^H and ^14^C.

### Biotin-Labeled Serotonin

Other studies used biotinylated serotonin for transglutaminase reactions (Watts et al., 2009). However, when biotin is linked to the amino group of serotonin, TG can not anymore use the adduct as substrate. Moreover the molecule gets very large and can not enter cells anymore. Therefore biorthogonal labeling was developed which used a small alkyne-functionalized serotonin, which can be taken up in cells for the transglutaminase reaction (Lin et al., 2013, 2014). Afterwards a biotin-residue is specifically linked by click-chemistry to this incorporated propargylated serotonin. The biotin-label then allows purification and detection of the serotonylated protein on Western blots and by mass spectrometry (Lin et al., 2013, 2014; Farrelly et al., 2019).

### Serotonin Antibodies

Antibodies against serotonin are produced by injection of animals with conjugates of bovine serum albumin or other proteins with serotonin. Therefore they are well-suited to detect serotonin when it is bound to proteins and can be used in Western blots and for immunoprecipitation (Guilluy et al., 2007, 2009; Dai et al., 2008; Paulmann et al., 2009; Watts et al., 2009; Liu et al., 2011; Abdala-Valencia et al., 2012; Wang et al., 2012, 2016; Penumatsa K. et al., 2014; Penumatsa K. C. et al., 2014; Penumatsa et al., 2017; Cui and Kaartinen, 2015; Ivashkin et al., 2015, 2019; Al-Zoairy et al., 2017; Mi et al., 2017). The problem in this case is cross reactivity with other non-serotonylated proteins which can rarely be excluded with any antibody.

### Two-Dimensional Gel Electrophoresis

Guilluy et al. (2007) have used two-dimensional gel electrophoresis to show that fibronectin changes its isoelectric point when serotonylated. They proved the identity of the spot by Western blot with an anti-serotonin antibody.

### Serotonin as Competitor

Since it was technically difficult to detect protein-bound serotonin some groups used mono-dansylcadaverine (MDC) or 5-(biotinamido) pentylamine as transglutaminase substrates, which can be easily detected in protein gels by fluorescence or by anti-biotin antibodies, respectively (Hummerich and Schloss, 2010; Cui and Kaartinen, 2015; Hummerich et al., 2015; Farrelly et al., 2019). When the binding of these substrates to proteins was inhibited by serotonin, they concluded that serotonin can also be bound to the same proteins. However, this method provides only indirect evidence and needs independent verification. Furthermore, MDC is also often used as inhibitor of transglutaminases.

### Mass Spectrometry

The most reliable evidence for the existence of serotonylation comes from mass spectrometry of proteins. By this method peptide fragments of proteins were discovered which had exactly the increase in molecular mass as predicted when serotonin was attached to a Gln residue (Farrelly et al., 2019). In most cases also the Gln residue could be distinguished. For this method serotonylated peptides are enriched by immunoprecipitation with serotonin-antibodies and therefore quantitative measurements of serotonylation are hardly possible.

## Substrates for Serotonylation

### Extracellular Proteins

The first described function of protein serotonylation concerned extracellular proteins on hyper-stimulated platelets, in particular fibrinogen, von Willebrand factor, and fibronectin (Dale et al., 2002; Table 1). The covalently bound serotonin residues on these proteins serves as glue to retain the proteins on the surface of these so-called coated platelets (Dale, 2005). The authors provided evidence that factor XIIIa is the main transglutaminase involved, but also TG2 could not be excluded. Serotonylation of fibronectin was later confirmed in a glial cell line by binding of MDC which could be competed by serotonin (Hummerich and Schloss, 2010) and by radioactive serotonin incorporation (Hummerich et al., 2012). Serotonylation of fibronectin led to an increased protein accumulation in the extracellular matrix of these cells (Hummerich et al., 2012). Incorporation of propargyl serotonin followed by mass spectrometry confirmed that Gln34, Gln38 and Gln40 are serotonylated in fibronectin (Lin et al., 2013) as had been shown when fibronectin was labeled with MDC (Hoffmann et al., 2011). Interestingly already in 1976 fibronectin had been discovered as factor XIIIa substrate using MDC (Mosher, 1976; Mosher et al., 1980).

**TABLE 1:** List of serotonylated proteins.

**Protein**	**Cell type**	**References**
Fibronectin	Platelets	Dale et al., 2002
	Vascular smooth muscle	Penumatsa K. C. et al., 2014
	Glioma	Hummerich and Schloss, 2010
	Osteoblasts	Cui and Kaartinen, 2015
Fibrinogen	Platelets	Dale et al., 2002
	Glioma	Hummerich et al., 2015
rab4, rhoA	Platelets	Walther et al., 2003
rhoA	Vascular smooth muscle	Guilluy et al., 2007; Wang et al., 2012
rab3a, rab27a	Pancreatic β-cells	Paulmann et al., 2009
rab4	Skeletal muscle cells	Al-Zoairy et al., 2017
rac1	Neurones	Dai et al., 2008
ras	Colorectal adenocarcinoma	Lin et al., 2013
Histones	Neurones	Farrelly et al., 2019
Actins, myosins	Vascular smooth muscle	Watts et al., 2009
Akt	Vascular smooth muscle	Penumatsa K. et al., 2014
SERCA2a	Cardiomyocytes	Wang et al., 2016

In contrast, the serotonylation of fibronectin in osteoblasts by factor XIIIa inhibits its crosslinking with other proteins and thereby destabilizes the matrix and reduces mineralization (Cui and Kaartinen, 2015).

The group of Fanburg has shown in several publications that upregulated TG2 in pulmonary arterial hypertension (PAH) serotonylates fibronectin in pulmonary arteries and thereby contributes to the disease pathogenesis (Liu et al., 2011; Penumatsa K. C. et al., 2014; Penumatsa and Fanburg, 2014; Liu et al., 2011).

### Small GTPases

When a certain Gln residue in small G-proteins [e.g., Gln61 in human Ras (Lin et al., 2013)] is serotonylated by TG2 the protein gets constitutively active (Walther et al., 2003; Aktories, 2011). Since there is yet no reverse reaction known inactivation can probably only be achieved by proteasomal degradation, however, there are hints that this degradation gets accelerated by serotonylation (Guilluy et al., 2007; Lin et al., 2013). We first discovered the serotonylation of small G-proteins in platelets where rab4 and rhoA serotonylation contributes to the effect of serotonin on platelet aggregation (Walther et al., 2003; Table 1). Serotonin has a dual role in platelets: it binds to 5-HT2A receptors on the plasma membrane and increases intracellular Ca^2+^ which activates TG2 and is taken up in the cytoplasm by the serotonin transporter (SERT) serving as substrate for TG2-mediated serotonylation. Serotonylation of small G-proteins probably by TG2 is also involved in insulin secretion by pancreatic beta cells. In these cells, serotonin promotes insulin secretion by covalently modifying rab3a and rab27a, which are essential small G-proteins in the secretory pathway of beta cells (Paulmann et al., 2009). Accordingly, TG2 deficient mice are glucose intolerant and show impaired insulin release (Bernassola et al., 2002). Also insulin sensitivity in muscle seems to be regulated by serotonin via serotonylation of small G-proteins. When L6 skeletal muscle cells are treated with serotonin, rab4 is serotonylated which leads to its activation and thereby to an increased glucose uptake (Al-Zoairy et al., 2017). This effect can be blocked by the TG2 inhibitor MDC.

The serotonin induced proliferative response of pulmonary arterial smooth muscle cells, which is involved in the pathogenesis of PAH is also mediated by the serotonylation of the small G-protein, rhoA (Guilluy et al., 2007, 2009; Wang et al., 2012). RhoA had already earlier been shown to be a substrate of TG2 at Gln63 using MDC for transamidation (Schmidt et al., 1998). Inhibition of serotonin uptake or of TG2 inhibits rhoA serotonylation, and cellular proliferation and contraction. Accordingly, blockers of TG2, SERT, and rhoA ameliorate PAH development (Guilluy et al., 2009; Wang et al., 2012, 2018).

In primary cortical neurons serotonin via 5-HT2A receptors stimulates TG2 which serotonylates Rac1 and thereby regulates spine density (Dai et al., 2008; Mi et al., 2017). Using propargylated serotonin, Lin et al. (2013) showed that also Ras gets serotonylated and thereby activated in different cell types.

### Histones

Already in 1996 it had been described that histone 3 can be modified with MDC at Gln5 by TG2 *in vitro* (Ballestar et al., 1996). However, only recently it was shown that *in vivo* serotonin is the amine covalently linked by TG2 to this residue of histone H3, using propargylated and radioactive serotonin as well as competition of MDC labeling by serotonin and mass spectrometry (Farrelly et al., 2019; Table 1). While Ballestar et al. (1996) had found also other histones in chicken nucleosomes to be transamidated *in vitro*, Farrelly et al. (2019) could not confirm these findings in human cells. Future studies need to clarify whether other histones than H3 can be serotonylated in certain species, cell types, or conditions. The serotonylation of Gln5 in histone H3 does not interfere with trimethylation on the neighboring Lys4 (H3K4me3) but exerts a permissive transcriptional activity in neuronal cells in culture and in the developing mouse brain. The reader for this novel histone modification is still unknown but it could be shown that the binding of the transcription factor TFIID to H3K4me3 bearing promoters and consequently transcription initiation gets facilitated by it (Farrelly et al., 2019). Based on these findings histone serotonylation became a novel epigenetic regulatory mechanism (Anastas and Shi, 2019; Fu and Zhang, 2019; Zhao et al., 2019; Zlotorynski, 2019). It remains to be established whether some of the phenotypes observed in mice lacking tryptophan hydroxylase 2 (TPH2) are depending on altered epigenetic control of gene expression due to the lack of brain serotonin (Alenina et al., 2009).

### Other Proteins

The signaling protein Akt has been revealed as TG2 substrate for serotonylation again in pulmonary arterial smooth muscle cells (Penumatsa K. et al., 2014; Table 1). Since TG2 is upregulated in these cells in PAH, Akt serotonylation may contribute to pulmonary artery remodeling in this disease.

In isolated cardiomyocytes high concentrations of serotonin induces the serotonylation of SERCA2a (Wang et al., 2016; Table 1). The physiological consequences of this modification are however, not clarified.

In cultured aortic smooth muscle cells contractile proteins such as actins and myosins can get serotonylated and the inhibition of TG2 by cystamine reduces serotonin induced contraction of these cells (Watts et al., 2009; Table 1). However, despite that mass spectrometry was performed the serotonylated Gln residues in these proteins were not reported which would have supported the specificity of the labeling method using biotinylated serotonin. Notwithstandingly, at least some of the discovered proteins, such as actin, were confirmed in other studies using propargylated serotonin or MDC and mass spectrometry in different cell types (Lin et al., 2014; Hummerich et al., 2015).

In allergic lung inflammation, the serotonin precursor, 5-hydroxytryptophan ameliorates the symptoms in mice and reduces the amount of serotonylated proteins in lung endothelium, as well as in cultured pulmonary endothelial cells (Abdala-Valencia et al., 2012). However, since anti-serotonin antibodies were used in immunohistochemistry, the serotonylated proteins were not identified.

Ivashkin et al. (2015, 2019) have published two studies in which they used anti-serotonin antibodies to detect serotonylated proteins in embryos of snails, sea urchins, mollusks, and zebrafish. TG2 inhibition by cystamine diminished the signals. In each case they found enrichment of these proteins in nuclei and several bands on Western blots but they did not identify the target proteins.

## Concluding Remarks and Future Perspectives

Compared to other covalent protein modifications, such as phosphorylation, serotonylation has only quite recently been discovered and not many functional consequences of it have been described. Serotonin in the cytoplasm is only available in a limited amount of cell types, which either generate their own serotonin by expressing one of the two tryptophan hydroxylases, TPH1 and TPH2, or import it employing SERT or other less specific transporter such as the Plasma Membrane Monoamine Transporter (PMAT, SLC29A4), and the Organic Cation Transporters OCT1 (SLC22A1), 2 (SLC22A2), or 3 (SLC22A3) (Walther and Bader, 2003; Jonker and Schinkel, 2004; Daws, 2009). Therefore serotonylation is expected not to occur in every cell type. However, other monoamines such as histamine, norepinephrine and dopamine are also substrates of TGs and may replace serotonin in certain cell types by also being used for “monoaminylation” (McConoughey et al., 2010; Walther et al., 2011; Muma and Mi, 2015). This kind of protein modification seem to be evolutionarily old, since it can be found from snails to vertebrates (Ivashkin et al., 2015, 2019) and uses a molecule, serotonin, which is present in nearly all organisms on earth (Turlejski, 1996; Walther et al., 2011; Csaba, 2015).

Still a lot of mechanistic questions remain open. Are all TGs involved in monoaminylation or mainly TG2 and, for extracellular targets, factor XIIIa? How specific are TGs for distinct Gln residues on selected target proteins and how is the specificity brought about? Are there any cofactors involved which confer specificity? Can TGs distinguish between monoamines or is just the concentration in the cell decisive about which monoamine is taken? In this respect, there are first data that TG2 has different affinities for histamine, norepinephrine and serotonin, serotonin being surprisingly the worst substrate (Clarke et al., 1959; Lai and Greenberg, 2013). And finally is there any enzymatic de-monoaminylation?

Despite the few examples of serotonylation which have been discovered so far, it has already been implicated in important physiological and pathophysiological processes. Probably it plays a major role in transcriptional regulation during development in several organisms by modulating histone-dependent epigenetic effects (Ivashkin et al., 2015, 2019; Farrelly et al., 2019). Moreover it contributes to the pathogenesis of PAH (Penumatsa and Fanburg, 2014; Wang et al., 2018) and therefore serotonin may be a valid therapeutic target for this disease (Matthes and Bader, 2018). Extracellular and intracellular serotonylation has been found to be involved in platelet aggregation and insulin secretion again offering therapeutic options (Walther et al., 2003; Dale, 2005; Paulmann et al., 2009). Since TG and serotonin have numerous additional pathophysiologically relevant functions (Berger et al., 2009; Daubert and Condron, 2010; McConoughey et al., 2010; Eckert et al., 2014; Lai et al., 2017; Matthes and Bader, 2018), some of them may be dependent on each other and based on serotonylation of yet unknown target proteins. Accordingly in drosophila and mouse neurons an induced increase of serotonin particularly in the cytoplasm leads to abnormalities reminiscent of neurodegenerative disorders (Daubert and Condron, 2010). In these models as well as in other comparable situations in which cytoplasmatic or nuclear serotonin is increased the signaling via serotonylation should be considered and may offer novel mechanistic insights and therapeutic options for a multitude of diseases.

## Author Contributions

The author confirms being the sole contributor of this work and has approved it for publication.

## Conflict of Interest

The author declares that the research was conducted in the absence of any commercial or financial relationships that could be construed as a potential conflict of interest.
